# Multimodal functional imaging of brown adipose tissue

**DOI:** 10.1194/jlr.ILR120001204

**Published:** 2020-11-28

**Authors:** Amanda D.V. MacCannell, John Wright, Jurgen E. Schneider, Lee D. Roberts

**Affiliations:** 1 Leeds Institute of Cardiovascular and Metabolic Medicine, University of Leeds, Leeds, United Kingdom; 2 PET Research Centre, Faculty of Health Sciences, University of Hull, Hull, United Kingdom

**Figure:**
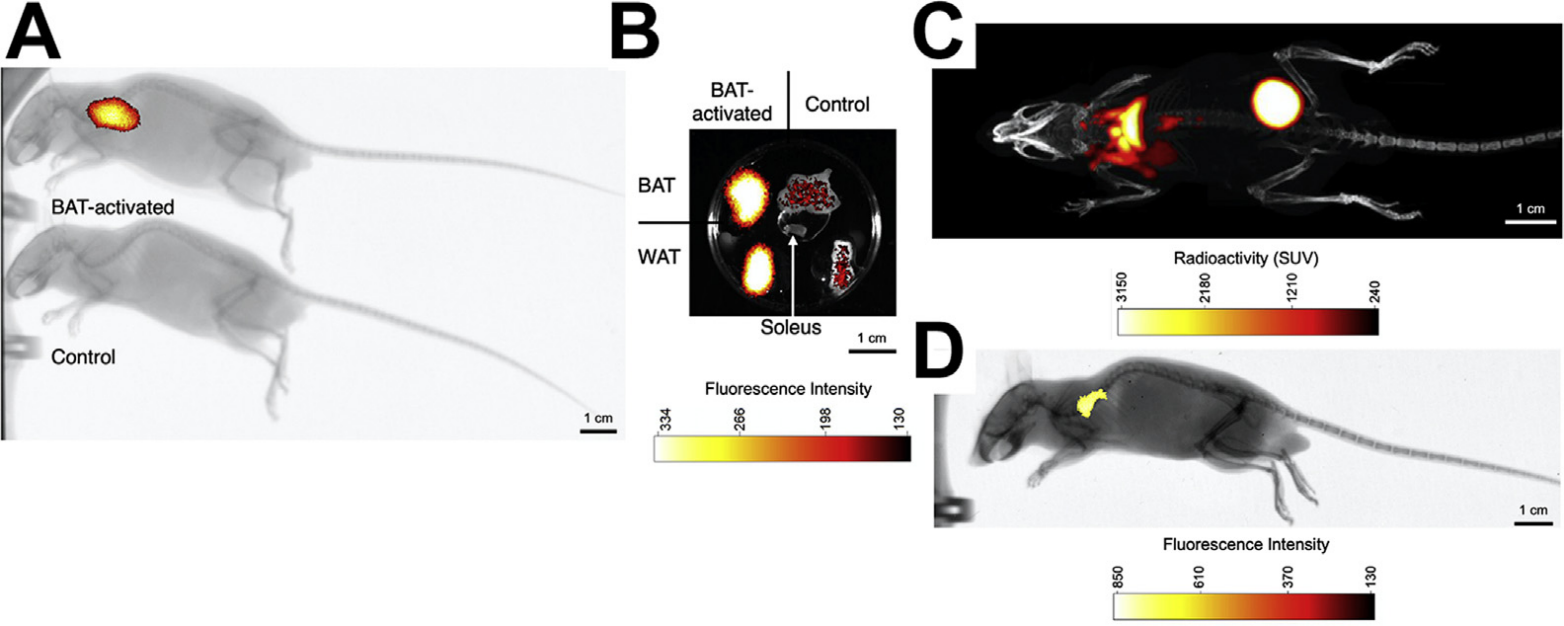

Brown adipose tissue (BAT) is a mitochondrial dense tissue capable of regulating body temperature and energy balance (1). BAT is a potential therapeutic target for metabolic diseases including obesity and type 2 diabetes (1). Determining in vivo BAT metabolic activity is a powerful tool in translational research. Positron emission tomography (PET) using ^18^F-fluorodeoxyglucose (FDG) is the standard technique for imaging BAT glucose uptake as a proxy for thermogenic activity (2). However, PET is limited by the requirement for radioisotope tracers, associated costs, and a lack of functionality to detect concurrent metabolic processes within the same animal. Multimodal imaging can overcome these limitations. We combined FDG PET with fluorescence optical imaging, a promising technique, not yet widely used in BAT studies (3). We induced BAT activity in C57BL6 mice with CL316,243, a highly specific beta 3-adrenoreceptor agonist, with 1 mg/kg subcutaneous injection for 3 days. We intravenously injected a commercially available fluorescent probe, RediJect 2-DG (100 μl), 3 h before imaging with an Xtreme II optical imaging system (Bruker, Ettlingen) in CL316,243-treated BAT-activated animals or saline-injected controls (panel A). Anatomical regions of interest were used in analysis of fluorescence optical imaging. Animals treated with beta 3-adrenoreceptor agonist had higher uptake of RediJect 2-DG in BAT, which we confirmed with ex vivo optical imaging of harvested tissues including BAT, subcutaneous white adipose tissue (WAT), and soleus muscle (panel B). Next, we compared RediJect 2-DG to FDG to determine if RediJect 2-DG was a suitable alternative to FDG and to establish the impact of co-injection. We co-injected RediJect 2-DG and FDG into a mouse with induced BAT activity. In succession, we imaged the same mouse with PET/computed tomography to detect the FDG (panel C) and then used optical imaging to detect the RediJect 2-DG (panel D). RediJect 2-DG optical imaging identifies increased activity in the BAT anatomical region as was observed with PET and validated ex vivo using optical imaging and gamma-counter biodistribution analysis. This study is an important step to progress onto wider multitracer work. Simultaneous co-injection of a radioisotope and fluorescent probe could expand current BAT in vivo imaging modalities and facilitate the future detection of multiple concurrent metabolic processes in a single animal.

**EQUIPMENT:** Albira Si PET/SPECT/CT (Bruker), Xtreme II optical imaging system (Bruker)

**REAGENTS:** XenoLight RediJect 2-DeoxyGlucosone (DG) (PerkinElmer), CL316,243 (Sigma)
